# Learning Phonemic Vowel Length from Naturalistic Recordings of Japanese Infant-Directed Speech

**DOI:** 10.1371/journal.pone.0051594

**Published:** 2013-02-20

**Authors:** Ricardo A. H. Bion, Kouki Miyazawa, Hideaki Kikuchi, Reiko Mazuka

**Affiliations:** 1 Department of Psychology, Stanford University, Stanford, California, United States of America; 2 Laboratory for Language Development, RIKEN Brain Science Institute, Wako-shi, Saitama, Japan; 3 Faculty of Human Sciences, Waseda University, Tokorozawa, Saitama, Japan; 4 Department of Psychology & Neuroscience, Duke University, Durham, North Carolina, United States of America; Baycrest Hospital, Canada

## Abstract

In Japanese, vowel duration can distinguish the meaning of words. In order for infants to learn this phonemic contrast using simple distributional analyses, there should be reliable differences in the duration of short and long vowels, and the frequency distribution of vowels must make these differences salient enough in the input. In this study, we evaluate these requirements of phonemic learning by analyzing the duration of vowels from over 11 hours of Japanese infant-directed speech. We found that long vowels are substantially longer than short vowels in the input directed to infants, for each of the five oral vowels. However, we also found that learning phonemic length from the overall distribution of vowel duration is not going to be easy for a simple distributional learner, because of the large base-rate effect (i.e., 94% of vowels are short), and because of the many factors that influence vowel duration (e.g., intonational phrase boundaries, word boundaries, and vowel height). Therefore, a successful learner would need to take into account additional factors such as prosodic and lexical cues in order to discover that duration can contrast the meaning of words in Japanese. These findings highlight the importance of taking into account the naturalistic distributions of lexicons and acoustic cues when modeling early phonemic learning.

## Introduction

Infants learning a language must discover which acoustic cues can be used to contrast the meaning of words. When learning English, the infant must discover that the words *bed* and *bad* differ mainly in vowel height. If learning Japanese instead, the infant must discover that the words/toko/(*bed*) and/toko:/(*travel*) differ only in the duration of their final vowels. In these examples, the contrast that is phonemic in English is not phonemic in Japanese and vice versa. The contrast between mid-open and near-open vowels is only relevant in English, while the contrast between short and long vowels is only relevant in Japanese.

In order for infants to learn which contrasts are relevant in their language, at least two requirements should be met. The first requirement is that contrastive sounds should be acoustically different from each other [1]. In the case of phonemic duration, long vowels should be significantly longer than short vowels. The second requirement is that the frequency distribution of the relevant acoustic cue should make the difference salient enough in the input. Infants should be able to learn that duration is contrastive in Japanese by determining the number of modes in the distribution of vowel durations without any knowledge about vowel identity [2], [3], [4]. In this paper, we examine these two requirements of phonemic learning by focusing on phonemic vowel length in Japanese. We first determine whether there are reliable differences in the duration of long and short vowels in Japanese infant-directed speech (IDS). We subsequently explore whether the frequency distribution of vowels from over 11 hours of naturalistic recordings provides enough evidence that duration is phonemic. We will argue that in order to investigate how infants may learn phonemic categories, it is crucial to take into account not only the acoustic cues associated with the category distinction, but also their naturalistic distribution in the input.

Previous studies suggest that there are reliable acoustic differences in the way Japanese mothers pronounce short and long vowels. Werker and colleagues recorded Japanese mothers' elicited production and reading of words such as/kido/and/ki:do/ [1]. They found a clear difference in the duration of the vowels in these minimal pairs, but no differences in other non-phonemic cues. These findings show that Japanese speech might contain acoustic cues that make language-specific learning possible. These results are promising, but they were based on the controlled recordings of nonce minimal pairs with vowels always occurring in closed syllables. The first goal of our study is to extend on these findings using naturalistic recordings, and including a much larger sample of real words from Japanese IDS.

Subsequent studies provided a specific account of how phonemic learning might occur. Vallabha and colleagues introduced a learning algorithm that learns vowel categories through analyses of the frequency distributions of acoustic cues in the input [2]. This model learned that duration is contrastive in Japanese without knowing in advance the number of vowel categories, and without being given any information about the phonemic duration of each vowel. However, several assumptions were made about the distribution of vowel duration. It was assumed that short and long vowels occurred with the same frequency in the input and were bimodally distributed. The second goal of this paper is not to challenge the importance Vallabha and colleagues model [2], but to explore whether these assumptions are met by naturalistic recordings of vowel duration in Japanese IDS.

Experimental studies emphasize the importance of looking at the frequency distribution of acoustic cues in the input. Adults and infants are sensitive to the shape of the distribution of sounds they hear [3], [4]. Infants could discriminate the sounds at the end of a continuum when they were previously familiarized with a bimodal distribution, but not when they were familiarized with a unimodal distribution instead. Whether infants can extract a distinction from the acoustic cue depends not only on the availability of the cues (i.e., short vowels being shorter than long vowels), but also on how often each of them occurs and whether they are present in different modes in the input.

In addition to differences in frequency, several other factors might complicate the learning of vowel duration in Japanese. In addition to phonemic status, the duration of a vowel can be influenced by its height [5], lexical stress [6], as well as contrastive stress and semantic novelty [7]. Vowel duration can also be influenced by final lengthening and speech rate [8]. These non-phonemic influences on vowel duration might make simple distributional analyses of the duration of vowels in the input particularly challenging. The third goal of this paper is to explore how different variables impact the duration of vowels in Japanese, comparing the influence of phonemic and non-phonemic factors.

In short, this study has three main goals: our first goal is to extend the findings of Werker and colleagues [1], comparing the duration of short and long vowels in naturalistic recordings of Japanese IDS in all syllables; our second goal is to explore whether the frequency distribution of vowel duration in Japanese allows for a simple distributional learning of phonemic length as the one implemented by Vallabha and colleagues [2]; our third goal is to explore the impact of phonemic and non-phonemic cues in the duration of vowels in Japanese. We will show that while there are reliable differences in the duration of short and long vowels in Japanese, differences in the frequency of these vowels and non-phonemic factors make the learning of vowel duration particularly challenging for simple distributional algorithms.

## Methods

### Participants

22 mothers, originally recorded for the RIKEN Japanese Mother-Infant Conversation Corpus [9], participated in this study. They were native speakers of Japanese from the Tokyo area, and had infants from 18 to 24 months of age. The mothers consented to have their recordings included as part of a corpus that would be analyzed and used for different purposes. We also recorded the same mothers talking with an adult experimenter in order to obtain parallel data on adult-directed speech (ADS).

### Procedure

The IDS recordings consisted of a total 11 hours of mothers interacting with their 18–24-month-old infant. It contained approximately 50,000 words.

### Acoustic analyses

The analyses were done using Praat [10]. The beginning and end of the vowels in each of the words from the original recordings was manually segmented and measured. For each vowel, we added information about phonemic length (short or long), height (high, mid, and low), and about whether the vowel was immediately followed by a word boundary or intonational boundary. From these recordings, we removed ADS fragments from the IDS recordings, we also removed singing, laughing, coughing, onomatopoeias, and fragments the transcriber could not understand.

The coding for the corpus was based on the scheme developed for the Corpus of Spontaneous Japanese developed by the National Institute of Japanese Language [11]. Transcription was done by a highly-trained phonetician, and double checked by a second expert phonetician. The main expert phoneticians who transcribed our corpus were members of the original CSJ team, and were thoroughly trained for the use of the coding scheme (for a detailed description of the coding scheme, see [11]).

Whether a vowel is long or short was decided on the basis of the lexical item intended by the speaker. If the transcriber hears the word/oka:san/(mother), there is no ambiguity on the phonemic duration of each of the three vowels. The first vowel is phonemically short (i.e., it contains one mora), the second vowel is long (i.e., it contains two morae), and the third vowel is short. The transcriber would then label each vowel as phonemically short or long, and mark its start and end on the audio file, from which duration could be subsequently computed. The same reasoning applies to vowel height, for which the transcriber would be aware that the first vowel in this word is mid, and the second and third vowels are low. Most words were clearly pronounced and could be easily identified. The supporting context (including video recordings of the conversation) also facilitated the recognition of most lexical items. In the rare cases in which the lexical item was not easily recognized, it was marked as such and not included in our analyses. Intonational boundaries were also coded by the same experts, using modified X-JToBI, following CSJ coding scheme.

## Results

### Are there reliable acoustic differences between short and long vowels in naturalistic recordings of Japanese IDS?

**Figure 1:**
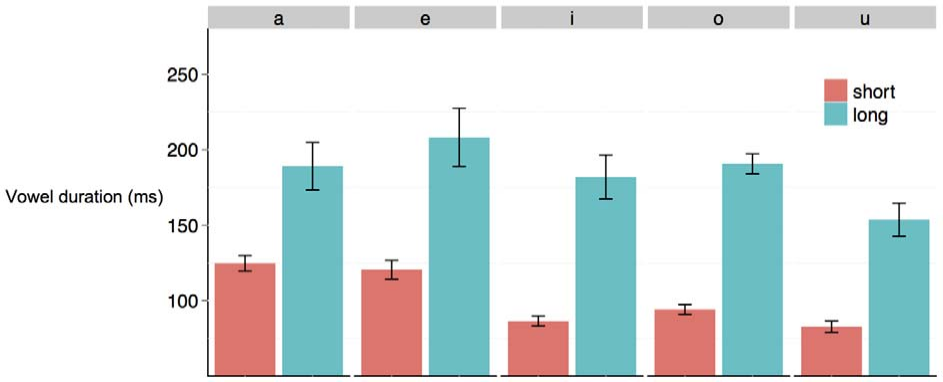
Mean duration of short and long vowels in the present Japanese IDS corpus. The difference in duration between short and long vowels is reliable and the effect size is large. The error bars represent the standard error of the mean for each vowel across participants.

In order to investigate whether there are reliable differences in the duration of short and long vowels in Japanese, the vowel duration data were analyzed in two independent analyses. The first analysis compares the average duration of short and long vowels for each of the five Japanese vowels (i.e., a, e, i, o, u). Towards this end, we computed for each mother a mean value for the duration of short and long vowels separately for each of the five oral vowels. We subsequently submitted these values to separate t-tests. As seen in Figure 1, long vowels are reliably longer than short vowels independently of the vowel produced. Cohen's *d* value ranged from 0.99 to 2.94, indicating that the effect size can be considered large [12], a, *t* (21)  = 6.77, *p*<0.001; e, *t* (21)  = 6.09, *p*<0.001; i, *t* (21)  = 8.66, *p*<0.001; o, *t* (21)  = 15.13, *p*<0.001; u, *t* (21)  = 9.25, *p*<0.001. These analyses confirmed that when the average duration of long and short vowels are compared, independently of the vowel produced, long vowels are in fact significantly longer than short vowels.

We then submitted the data to a hierarchical logistic regression, following Werker and colleagues [1], with vowel category entered as dependent value, vowel duration as independent variable, and speaker as a random effect. This model was highly significant in predicting vowel category (short vs. long) based on the duration of each vowel, *Estimate*  = 0.004, *Error*  = 0.0001, *z* = 37.66, *p*<0.001. These analyses were replicated separately for each speaker, using a simple logit model. For each individual speaker, the duration of her vowels in ms was a significant predictor of phonemic vowel length (for each of the 22 speakers, *z*>8.32, *p*<0.001). These analyses confirm that duration can be used to identify phonemic length in Japanese once listeners are aware that this acoustic cue is contrastive and that vowels should be classified as either short or long. The next section focuses on the situation in which learners are not aware of this distinction yet, and have to learn about it with no information about the phonemic status of vowels.

### Can simple distributional models account for the learning of phonemic duration in Japanese?

**Figure 2:**
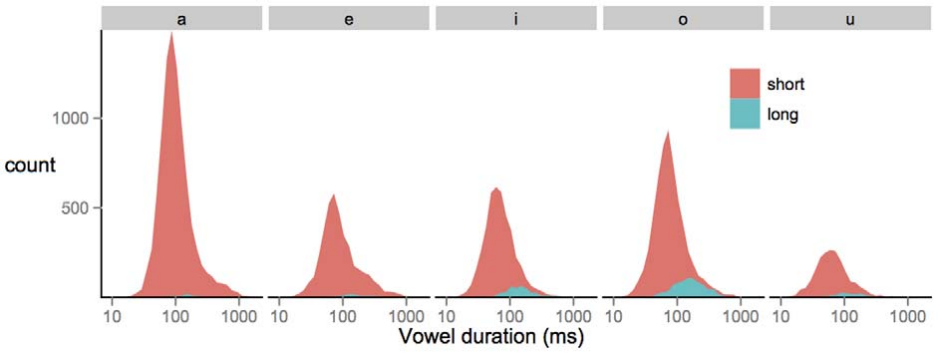
The frequency distribution of vowel duration in the present Japanese IDS corpus. Ninety-four percent of the vowels in our corpus are short, and there is complete overlap in the distribution of short and long vowels. This kind of input is problematic for simple distributional learning models.

In order to investigate whether simple distributional models can explain the learning of phonemic length in Japanese, we first plotted a histogram with all the vowels in our IDS sample, color-coded according to their phonemic duration. A visual inspection of Figure 2 reveals two findings: most of the vowels in this corpus are short, and there is complete overlap in the distribution of short and long vowels in the input for each of the five oral vowels. In fact, 94% of the vowels in Japanese are short (27,561), and only 6% of them are long (1,942), a pattern replicated for each of the 22 speakers individually. While Figure 1 suggests that phonemic learning might be easy in Japanese, Figure 2 suggests a very different picture.

Any mechanical approach that looks only for bimodal distributions in the input will not suffice, as the distribution of vowel duration in Japanese is unimodal, an observation confirmed statistically by Dip tests of unimodality. Only 6% of the vowels are long, and this low base rate is problematic for simple distribution learning models that are biased to minimize the number of phonemic categories in the input.

In order to confirm that this difference in base-rate between short and long vowels is not specific to our corpus, we also analyzed the duration of two additional ADS corpora. We recorded the same 22 mothers from our study talking to an adult experimenter. Using the same coding scheme, we found that 25,071 vowels were short, and only 2,018 vowels were long. In addition, we also analyzed the vowels on the CSJ [11]. Again, there were 449,494 short vowels, and only 48,032 long vowels. These additional analyses confirm our finding that over 90% of vowel tokens from spontaneously spoken Japanese are short. Kunnari and colleagues found a similar difference in base-rate between geminate and singleton consonants, with 93% of Japanese consonants consisting of a single mora (singleton) [13].

These analyses were all done on tokens, and therefore our next step was to investigate whether the same pattern of results is found when we look at the distribution of types in Japanese IDS. Towards this end, we first computed the mean duration for each vowel in each of the different word types in our corpus. There were a total of 1179 different words in our IDS corpus, with an average of 2.12 vowels per word, 2511 vowels in total. A total of 94% of these vowels are short, replicating the previous differences in base rate. And once more the distribution is unimodal, with no clear distinct modes in the input for short and long vowels.

### Do non-phonemic factors influence the duration of vowels in Japanese?

In this section, we evaluate which factors – in addition to phonemic length – might influence duration of vowels in Japanese. We ran a hierarchical mixed model, predicting vowel duration with information about phonemic length (short or long), position within intonational phrase (presence of IP boundary), position within the word (presence of word boundary), and vowel height (high, mid, and low). This statistical approach is appealing because it does not require us to average vowel duration across conditions, and it also takes into account the nested nature of our data (i.e., several vowels are produced by each of the speakers). For a review of the advantages of mixed-effect models, see [14].

We replicated the results from our previous analyses: Phonemic long vowels are longer than short vowels, as evidenced by the extremely high z scores (in mixed models, z scores greater than 2 are suggestive of a significant effect).

In addition, we also found that several other factors influenced the duration of vowels in Japanese: vowels are longer when followed by an intonational phrase boundary, shorter when followed by a word boundary that is not an IP boundary, and low vowels are longer than high vowels. We added interaction terms between phonemic vowel duration and the different factors that might impact the duration of vowels in Japanese. The only significant interaction was between word boundary and phonemic length, indicating that the impact of word boundaries in vowel duration is slightly greater for long vowels. But it is unlikely that the fact that 10% of vowels in infants' input is slightly more affected by the presence of word boundaries can serve as a reliable cue that vowel duration is contrastive in Japanese. In order to confirm the effect of vowel height, intonational boundary, and word boundary are observed specifically for the short or the long vowels, the same analysis (minus phonemic vowel duration) were carried out separately for short and long vowels. As the results in Table 1 show, all the factors remain highly significant in both analyses. We do not question whether infants can compensate for non-phonemic factors such as the presence of intonational or word boundaries and differences in vowel height. Yet, it is not clear how much this ability would help them discover that vowel duration is contrastive in Japanese. We tried several additional analyses in our dataset, including normalizing vowel duration into z scores for each speaker or taking into account the duration of the following and preceding vowels in our analyses, as well as computing separate distributions for vowels at intonational or word boundaries. These approaches and many others still resulted in a unimodal distribution due to the differences in base-rate between short and long vowels.

**Table 1:** Coefficients and standard errors for analyses of factors influencing vowel duration in Japanese.

	all vowels			short vowels			long vowels		
	Estimate	Error	*z*	Estimate	Error	*z*	Estimate	Error	*z*
Intercept	155.88	4.48	34.65	55.81	3.86	14.46	180.97	12.97	13.91
Phonemic duration	98.41	2.41	40.68*						
IP boundary	73.28	1.54	47.54*	72.88	1.53	24.34*	73.16	8.50	8.60*
Word boundary	22.87	1.41	16.14*	18.94	1.43	13.23*	63.71	7.054	9.04*
Height	18.66	0.78	23.72*	18.97	0.79	24.34*	15.48	5.21	2.97*
IP x Dur	0.04	6.58	0.07						
Word x Dur	50.47	5.48	9.20*						
Height x Dur	6.93	4.02	1.72						

Note. Non-phonemic factors such as the presence of intonational or word boundaries, and vowel height, influence vowel duration in Japanese, for both short and long vowels.

## Discussion

This study aimed at exploring two often conflated requirements of early speech learning: (i) Sounds that contrast the meaning of words differ in their acoustic characteristics, and (ii) acoustically different sounds appear in easily detectable bimodal distributions in the input that allow for simple distributional learning of sound categories. Using phonemic vowel duration in Japanese as a case study, we analyzed the vowels produced during over 11 hours of naturalistic recordings. Our main conclusion is that in order to investigate how infants may learn phonemic categories from the input, it is critical to take into account not only the acoustic cues associated with the category distinction, but also their distribution. By looking at the natural distribution of vowels, we observed that most of the vowels in our corpus are short, and this difference in base-rate should make learning difficult for simple distributional models that are biased to minimize the number of categories in the input.

We first demonstrated that there are reliable differences in the duration of short and long vowels in Japanese. Importantly, these analyses extend the findings of Werker and colleagues [1] with naturalistic recordings, vowels in different word positions, and a much larger sample.

However, when we looked at the natural distribution of vowels in our corpus, we found that 94% of vowels are short and that the combined distribution of short and long vowels is unimodal. In addition, we found that the duration of vowels is influenced by non-phonemic factors such as the presence of intonational phrase or word boundaries as well as vowel height. These sources of variation are not phonemic, and they might make it even harder for infants to discover that duration is contrastive in their language.

Not surprisingly, Japanese infants take a long time to show evidence of sensitivity to phonemic vowel length. Japanese 4-month-old infants fail to discriminate the contrast between short and long vowels (e.g., /mana/vs./ma:na/), while 9.5-month-olds show sensitivity to the same distinction. By contrast, 4-month-olds showed sensitivity to a vowel quality change (e.g., /mana/vs./mina/, [15]). Additional studies suggest that it is not until 18 months of age that Japanese infants perceive vowel duration as a phonological cue, rather than as an acoustic-phonetic cue [16]. The weak evidence for phonemic duration we observed in naturalistic recordings of Japanese IDS might explain why duration is such a difficult cue to learn in Japanese.

How do Japanese infants learn that duration is contrastive in their language despite scarce evidence in the input? The idea that word contexts could help in learning phonetic categories even before kids know what many words mean is raised in [17], and this idea is expanded, with infant-directed speech examples, in [18]. Recent computational work called attention to the fact that overlapping phonetic categories can be learned more easily by using word-level contextual information [19], [20]. While early work assumed the need of bimodal distributions in the input [2], newer models demonstrated that vowel categories can be learned better when learned in conjunction with a lexicon by learners who are biased to minimize the number of categories and words they are learning [19].

The model proposed by Feldman and colleagues [19], [20] is promising, integrating biases and learning from different levels of linguistic description. This kind of joint learning strategy might be expanded to take into account contextual effects and non-phonemic factors. In addition, the model should also take base rates into account. That is, the conditional probability of encountering a phonemically long vowel given a duration should take into account the prior probability that the vowel has that duration due to both phonemic and non-phonemic factors, and the total probability of a vowel being phonemically long (e.g., in this case, a mere 6%).

Alternatively, the learning of phonemic length in Japanese can follow an entirely different developmental trajectory. Initially, Japanese infants might assume that duration is not contrastive in their language, based on the observed distribution of vowel duration in the input. Because of differences in base rate, classifying all vowels as short would already lead to over 94% of categorization accuracy. With language experience, infants become better at accounting for variation in duration due to the presence of intonational phrase and word boundaries, as well as differences in vowel height. Infants might have powerful normalization algorithms already in place, but they cannot yet account for the variation in vowel duration due to phonemic length, and perceive these tokens as atypical, longer exemplars of short vowels. At around 18 months of age, when infants have experienced many of these atypical exemplars and cannot yet explain why these vowels are considerably longer, infants might gain the insight that duration can contrast the meaning of words in their language.

And they might also benefit from the presence of minimal pairs. After all, the words/obasan/(*aunt*) and/oba:san/(*grandmother*) refer to different things and differ only in their duration. Importantly, this difference cannot be accounted by contextual factors. We observed over 60 minimal pairs in our corpus, and these minimal pairs might help infants learn that duration is contrastive in their language. Our hypothesis that infants initially treat all vowels as short is supported by experimental work showing asymmetries in Japanese infants' detection of changes from short to long vowels and from long to short vowels [15]. Future work should test the hypotheses of these different learning models on the actual distribution of lexical items and acoustic cues in the input to children.
